# Complete Genome Sequence of *Lysinibacillus* sp. Strain SGAir0095, Isolated from Tropical Air Samples Collected in Singapore

**DOI:** 10.1128/MRA.00604-19

**Published:** 2019-09-19

**Authors:** Anthony Wong, Ana Carolina M. Junqueira, Akira Uchida, Rikky W. Purbojati, James N. I. Houghton, Caroline Chénard, Megan E. Clare, Kavita K. Kushwaha, Alexander Putra, Nicolas E. Gaultier, Balakrishnan N. V. Premkrishnan, Vineeth Kodengil Vettath, Cassie E. Heinle, Daniela I. Drautz-Moses, Stephan C. Schuster

**Affiliations:** a Singapore Centre for Environmental Life Sciences Engineering, Nanyang Technological University, Singapore; b Departamento de Genética, Instituto de Biologia, Universidade Federal do Rio de Janeiro, Rio de Janeiro, Brazil; University of Maryland School of Medicine

## Abstract

Lysinibacillus sp. strain SGAir0095 was isolated from tropical air samples collected in Singapore, and its complete genome was sequenced with a hybrid strategy using single-molecule real-time sequencing and short reads. The genome consists of one chromosome of 4.14 Mbp and encompasses 3,885 protein-coding genes, 39 rRNAs, and 101 tRNAs.

## ANNOUNCEMENT

Lysinibacillus spp. are rod-shaped, Gram-positive bacteria belonging to the family *Bacillaceae*. *Lysinibacillus* was first proposed as a genus distinct from Bacillus by Ahmed et al. (1) in 2007, as its peptidoglycan was found to contain lysine, aspartic acid, alanine, and glutamic acid. Since then, the reclassification of several *Bacillus* species to *Lysinibacillus* has been proposed (2–4). *Lysinibacillus* spp. have been found to be associated with plants (5–7) and also have been isolated from air in Makkah, Saudi Arabia (8), which contrasts with its natural soil habitat (1). Here, we report the complete genome sequence of another *Lysinibacillus* sp. collected from the air, which may aid future metagenomics studies and provide insight into its presence in the air microbiome.

*Lysinibacillus* sp. strain SGAir0095 was isolated from air collected in Singapore (global position system coordinates 1.347654N, 103.685240E) using an Andersen type air sampler (IUL S.A., Spain). The air was impacted onto marine agar (Becton Dickinson, USA), and subsequent isolation of the colonies was done on Trypticase soy agar (TSA; Becton Dickinson) at 30°C. Prior to DNA extraction, a single colony was cultured in lysogeny broth (Merck, USA) at 30°C overnight. Genomic DNA was purified using the Wizard genomic DNA purification kit (Promega, USA), following the manufacturer’s recommended protocol. Library preparation was performed with the SMRTbell template prep kit 1.0 (Pacific Biosciences, USA), followed by single-molecule real-time (SMRT) sequencing on the Pacific Biosciences RS II platform. The assembly was further supplemented with a 300-bp paired-end run to generate short reads on a MiSeq platform (Illumina, USA). The whole-genome shotgun libraries of short fragments were constructed using the TruSeq Nano DNA library preparation kit (Illumina), following the manufacturer’s protocol.

Subsequent analysis was performed using default software parameters, unless otherwise specified. Quality control was done with the PreAssembler filter version 1 from the Hierarchical Genome Assembly Process (HGAP) version 3 (9) and with Cutadapt version 1.8.1 (10) for PacBio and MiSeq reads, respectively. A total of 109,403 subreads from SMRT sequencing with an *N*_50_ length of 10,466 bp were used for *de novo* assembly using HGAP version 3 (9) implemented in the PacBio SMRT Analysis package version 2.3.0. The assembly was polished using Quiver (9) and error corrected using Pilon version 1.16 (11) and 716,265 MiSeq paired-end reads. The final assembly consisted of a circularized 4,142,194-bp chromosomal contig, with a coverage of 191.716-fold and a G+C content of 36.68%. Species evaluation was also performed with Microbial Species Identifier based on the genome-wide average nucleotide identity (ANI) method (12) using ANICalculator, with default parameters, against a database of 6,387 bacterial RefSeq genomes created using text filter for: “type, synonym type, proxytype” and subsequent “getorf -find 3” option. The closest identified species was Lysinibacillus sinduriensis BLB-1 (82.74%), with an alignment fraction of 38%. However, via 16S rRNA sequencing, the closest hit was *Bacillus* sp. strain B13 (99.47%).

The genome was annotated using the NCBI’s Prokaryotic Genome Annotation Pipeline (PGAP) version 4.4 (13). A total of 4,083 genes were predicted, consisting of 3,885 protein-coding genes, 13 copies each of 5S, 16S, and 23S rRNAs, 101 tRNAs, 6 noncoding RNAs, and 52 pseudogenes. Using Rapid Annotations using Subsystems Technology (RAST) (14–16) with the ClassicRAST annotation scheme with the “fix frameshift” option set to “yes,” functional annotation revealed that 402 and 325 genes were associated with amino acids and their derivatives and carbohydrate metabolism, respectively. These genes may play a role in the altered peptidoglycan that *Lysinibacillus* spp. possess. Interestingly, stress response- and dormancy-related genes (123 and 78 genes, respectively) were also predicted which may allow *Lysinibacillus* sp. strain SGAir0095 to remain dormant during transport in air.

### Data availability.

The complete genome sequence of *Lysinibacillus* sp. SGAir0095 has been deposited in DDBJ/EMBL/GenBank and the Sequence Read Archive (SRA) under the accession number CP028083 and numbers SRR8894403 and SRR8894404, respectively.
